# Are RNA networks scale-free?

**DOI:** 10.1007/s00285-019-01463-z

**Published:** 2020-01-16

**Authors:** P. Clote

**Affiliations:** Department of Biology, Boston College, Chestnut Hill, MA 02467 USA

**Keywords:** RNA secondary structure, Scale-free network, Small-world network, Dynamic programming, 62G32, 62G07, 68W99, 05C82, 68R05

## Abstract

A network is *scale-free* if its connectivity density function is proportional to a power-law distribution. It has been suggested that scale-free networks may provide an explanation for the robustness observed in certain physical and biological phenomena, since the presence of a few highly connected *hub* nodes and a large number of small-degree nodes may provide alternate paths between any two nodes on average—such robustness has been suggested in studies of metabolic networks, gene interaction networks and protein folding. A theoretical justification for why many networks appear to be scale-free has been provided by Barabási and Albert, who argue that expanding networks, in which new nodes are preferentially attached to highly connected nodes, tend to be scale-free. In this paper, we provide the first efficient algorithm to compute the connectivity density function for the ensemble of all homopolymer secondary structures of a user-specified length—a highly nontrivial result, since the exponential size of such networks precludes their enumeration. Since existent power-law fitting software, such as powerlaw, cannot be used to determine a power-law fit for our exponentially large RNA connectivity data, we also implement efficient code to compute the maximum likelihood estimate for the power-law scaling factor and associated Kolmogorov–Smirnov *p* value. Hypothesis tests strongly indicate that homopolymer RNA secondary structure networks are not scale-free; moreover, this appears to be the case for real (non-homopolymer) RNA networks.

## Introduction

The *connectivity* (or *degree*) of a node *v* in a network (or undirected graph) is the number of nodes (or neighbors) of *s*, connected to *v* by an edge. A network is said to be *scale-free* if its connectivity function *N*(*k*), which represents the number of nodes having degree *k*, satisfies the property that $N(a\cdot k) = b\cdot N(x)$, the unique solution of which is a *power-law* distribution, which by definition satisfies $N(k) \propto {k^{-\alpha }}$ for some scaling factor $\alpha >1$ (Newman 2006). Scale-free networks contain a few nodes of high degree and a large number of nodes of small degree, hence may provide a reasonable model to explain the robustness^1^ often manifested in biological networks—such robustness or resilience must, of course, be present for life to exist.

Barabasi and Albert (1999) analyzed the emergence of scaling in random networks, and showed that two properties, previously not considered in graph theory, were responsible for the power-law scaling observed in real networks: (1) networks are not static, but grow over time, (2) during network growth, a highly connected node tends to acquire even more connections—the latter concept is known as *preferential attachment*. In Barabasi and Albert (1999), it was argued that preferential attachment of new nodes implies that the degree *N*(*k*) with which a node in the network interacts with *k* other nodes decays as a power-law, following $N(k) \propto k^{-\alpha }$, for $\alpha >1$. This argument provides a plausible explanation for why diverse biological and physical networks appear to be scale-free. Indeed, various publications have suggested that the the following biological networks are scale-free: protein–protein interaction networks (Ito et al. 2000; Schwikowski et al. 2000), metabolic networks (Ma and Zeng 2003), gene interaction networks (Tong et al. 2004), yeast co-expression networks (Van Noort et al. 2004), and protein folding networks (Bowman and Pande 2010).

*How scale-free are biological networks?*

The validity of a power-law fit for previously studied biological networks was first called into question in Khanin and Wit (2006), where 10 published data sets of biological interaction networks were shown *not* to be fit by a power-law distribution, despite published claims to the contrary. Estimating an optimal power-law scaling factor by maximum likelihood and using $\chi ^2$ goodness-of-fit tests, it was shown in Khanin and Wit (2006) that not a single one of the 10 interaction networks had a nonzero probability of being drawn from a power-law distribution; nevertheless, some of the interaction networks could be fit by a *truncated* power-law distribution. The data analyzed by the authors included data from protein–protein interaction networks (Ito et al. 2000; Schwikowski et al. 2000), gene interaction networks determined by synthetic lethal interactions (Tong et al. 2004), metabolic interaction networks (Ma and Zeng 2003), etc.

In Clauset et al. (2009), 24 real-world data sets were analyzed from a variety of disciplines, each of which had been conjectured to follow a power-law distribution. Estimating an optimal power-law scaling factor by maximum likelihood and using goodness-of-fit tests based on likelihood ratios and on the Kolmogorov–Smirnov statistic for non-normal data, it was shown in Clauset et al. (2009) that some of the conjectured power-law distributions were consistent with claims in the literature, while others were not. For instance, Clauset et al. (2009) found sufficient statistical evidence to reject claims of scale-free behavior for earthquake intensity and metabolic degree networks, while there was insufficient evidence to reject such claims for networks of protein interaction, Internet, and species per genus.

It is possible to come to opposite conclusions, depending on whether $\chi ^2$ or Kolmogorov–Smirnov (KS) statistics are used to test the hypothesis whether a network is scale-free, i.e. follows a (possibly truncated) power-law distribution. Indeed, Khanin and Wit (2006) obtained a *p* value of $<10^{-4}$ for $\chi ^2$ goodness-of-fit for a truncated power-law distribution for the protein–protein interaction data from Ito et al. (2000), while Clauset et al. (2009) obtained a *p* value of 0.31 for KS goodness-of-fit for a truncated power-law for the same data.

In this paper, we introduce the first efficient algorithm to compute the exact number of homopolymer RNA secondary structures having *k* neighboring structures, for each value of *k*, that can be reached by adding or deleting one base pair. Since there are exponentially many secondary structures, our $O(n^5)$ time and $O(n^3)$ space algorithm uses dynamic programming. By applying the Kolmogorov–Smirnov test, we then show that homopolymer RNA secondary structure networks are not scale-free. We also provide evidence that the same is true for real RNA networks. Prior to this paper, only fragmentary results were possible by exhaustively enumerating all secondary structures having free energy within a certain range obove the minimum free energy (Wuchty 2003).

Our work investigates properties of the ensemble of RNA secondary structures, considered as a network, and so extends results of Clote (2015), which described a cubic time dynamic programming algorithm to compute the expected network degree. The RNA connectivity algorithm described in Sect. 2.3 is completely unrelated to that of Clote (2015), and allows one to compute all finite moments, including mean, variance, skew, etc.

The plan of the remaining paper is as follows. Section 2 presents a brief summary of basic definitions, followed by a description of an efficient dynamic programming algorithm to determine the absolute [resp. relative] frequencies *N*(*k*) [resp. *p*(*k*)] for secondary structure connectivity of a given homopolymer, which allows non-canonical base pairs. Section 3 presents the statistical methods used to both fit RNA connnectivity data to a power-law distribution and to perform a goodness-of-fit test using Kolmogorov–Smirnov distance. Section 4 shows that RNA networks are not scale-free, by performing (computationally efficient) Kolmogorov–Smirnov bootstrapping tests. Section 5 presents concluding remarks, while the Appendix presents data that suggests that RNA networks satisfy a type of preferential attachment. The rigorous proof that RNA networks satisfy modified form of preferential attachment is suppressed for reasons of space, but is available in the preprint (Clote 2018).

## Computing degree frequency

Section 2.1 presents basic definitions and notation used; Sect. 2.2 presents an algorithm to compute the frequency of each degree less than *K* in the ensemble of all secondary structures with run time $O(K^2 n^4)$ and memory requirements $O(K n^3)$. Section 2.3 presents a more efficient algorithm, with run time $O(K^2 n^3)$ and memory requirements $O(K n^2)$, for the special case of a homopolymer, in which all possible non-canonical base pairs are permitted. We implemented both algorithms in Python, cross-checked for identical results, and call the resulting code RNAdensity. Since this paper is a theoretical contribution on network properties, we focus only on homopolymers and do not present the details necessary to extend the algorithm of Sect. 2.2 to non-homopolymer RNA, where base pairs are required to be Watson–Crick or GU wobble pairs—such an algorithm is possible to develop, using ideas of Sect. 2.2; however, since the resulting complexity is formidible, with $O(n^9)$ time and $O(n^7)$ space requirements, and since there are no obvious applications, we do not pursue such an extension.

### Preliminaries

A secondary structure for a length *nhomopolymer* is a set *s* of base pairs (*i*, *j*), such that (1) there exist at least $\theta$ unpaired bases in every hairpin, where $\theta$ is usually taken to be 3, though sometimes 1 in the literature, (2) there are no basd triples, so for $(i,j), (k,\ell ) \in s$, if $\{ i,j \} \cap \{k,\ell \} \ne \emptyset$, then $i=k$ and $j=\ell$, (3) there do not exist base pairs $(i,j), (k,\ell ) \in s$, such that $i<k<j<\ell$; i.e. a secondary structure is a type of outerplanar graph, where each base pair $(i,j) \in s$ satisfies $j-i>\theta$. The *free energy* of a homopolymer secondary structure *s* is defined to be $-1$ times the number |*s*| of base pairs in *s* [Nussinov–Jacobson energy model (Nussinov and Jacobson 1980)]. Since entropic effects are ignored, this is not a real free energy; however it allows us to use the standard notation “MFE” for ‘minimum free energy’. Note that the MFE structure for a length *n* homopolymer has $\lfloor \frac{n-\theta }{2} \rfloor$ many base pairs.

For a given RNA sequence, consider the exponentially large network of all its secondary structures, where an undirected edge exists between any two structures *s* and *t*, whose base-pair distance equals one—in other words, for which *t* is obtained from *s* by either removing or adding one base pair. The connectivity (or degree) of a node, or structure, *s* is defined to be the number of secondary structures obtained by deleting or adding one base pair to *s*—this corresponds to the so-called $MS_1$ move set (Flamm et al. 2000). At the end of the paper, we briefly consider the $MS_2$ move set, where the degree of a structure *s* is defined to be the number of secondary structures obtained by adding, deleting or *shifting* one base pair (Bayegan and Clote 2015). The $MS_1$ [resp. $MS_2$] connectivity of the MFE structure for a homopolymer of length *n* is $\lfloor \frac{n-\theta }{2} \rfloor$ [resp. $\lceil \frac{n-\theta }{2} \rceil$]. *ConnectivityN*(*k*) is defined to be the *absolute* frequency of degree *k*, i.e. the number of secondary structures having exactly *k* neighbors, that can be obtained by either adding or removing a single base pair. The *degree densityp*(*k*) is defined to be the probability density function (PDF) or *relative* frequency of *k*, i.e. the proportion $p(k) = \frac{N(k)}{Z}$ of all secondary structures having *k* neighbors, where *Z* denotes the total number of secondary structures for a given homopolymer. A network is defined to be *scale-free*, provided its degree frequency *N*(*k*) is proportional to a power-law, i.e. $N(k) \propto k^{-\alpha }$ where $\alpha >1$ is the *scaling factor*.

### Computing the degree density

In this section, we describe a novel dynamic programming algorithm to compute the $MS_1$*degree densityp*(*k*) for the network of secondary structures for a homopolymer of length *n*. Note first that the empty structure $s_{\emptyset }$ of length *n* has

1 $$\begin{aligned} \text{ degree }(s_{\emptyset })&= \frac{(n-\theta )(n-\theta -1)}{2} \end{aligned}$$

many neighbors, each obtained by adding a base pair. Indeed,

$$\begin{aligned} \text{ degree }(s_{\emptyset })&= \sum _{i=1}^{n-\theta -1} \sum _{j=i+\theta +1}^n 1 = \sum _{i=1}^{n-\theta -1} [n-(i+\theta +1)+1] \\&= \sum _{i=1}^{n-\theta -1} (n-i-\theta ) = (n-\theta )(n-\theta -1) - \sum _{i=1}^{n-\theta -1} i \\&= \frac{(n-\theta )(n-\theta -1)}{2} \end{aligned}$$

Using a simple induction argument, Eq. (1) implies that for all values of *n*, the maximum possible degree, $\text{ maxDegree }(n)$, of a secondary structure for the length *n* homopolymer is $\frac{(n-\theta )(n-\theta -1)}{2}$

Let *N*(*i*, *j*) denote the number of secondary structures on interval [*i*, *j*], computed the following simple recurrence relation from Stein and Waterman (1978): for $1\le i\le j \le i+\theta \le n$, set $N(i,j)=1$, and for $i+\theta +1 \le j \le n$ set

2 $$\begin{aligned} N(i,j)&= N(i,j-1) + N(i+1,j-1) + \sum _{r=i+1}^{j-\theta -1} N(i,r-1) \cdot N(r+1,j-1) \end{aligned}$$

or more simply

3 $$\begin{aligned} N(m)&= \left\{ \begin{array}{ll} 1 &{}\quad \text{ if } \, 1 \le m \le \theta +1 \\ N(m-1) + N(m-2) + \displaystyle \sum _{r=\theta }^{m-3} N(m-r-2) \cdot N(r) &{}\quad \text{ if } \, \theta +2 \le m \le n \end{array} \right. \end{aligned}$$

Although Eq. (2) requires $O(n^3)$ time and $O(n^2)$ space, it can trivially be extended to compute the number of secondary structures for an arbitary RNA sequence $a_1,\ldots ,a_n$, where base pairs are either Watson–Crick or wobble pairs. If no such extension is necessary, then the recurrence relation Eq. (3), first given in Stein and Waterman (1978), requires $O(n^2)$ time and *O*(*n*) space, hence is more efficient by a factor of *n*. In a similar fashion, the recurrence relations (5–12) and pseudocode in Sect. 2.2 are given in a form that allows an extension (not given here) to the general case of computing the degree density for the ensemble of secondary structures of a given RNA sequence $a_1,\ldots ,a_n$. The top-level pseudocode given in Algorithm 1 requires $O(n^6)$ time and $O(n^4)$ storage; however, in the next section, we improve this by a factor of *n*, both in time and space requirements.

Suppose that every hairpin loop is required to have at least $\theta \ge 1$ unpaired positions; i.e. if (*i*, *j*) is a base pair, then $i+\theta +1 \le j$. As described in the Eqs. (5–12) for Base Cases A–D, let *Z*(*i*, *j*, *k*, *h*, *v*) denote the number of secondary structures on the interval [*i*, *j*], for $1 \le i \le j \le n$ for the homopolymer model, that have exactly *k* neighbors, and for which there are exactly *h* unpaired positions (or *holes*) in $[i,j-\theta -1]$, and for which there are exactly $v \in [0,\theta +1]$ visible positions among $j-\theta ,j-\theta +1,\ldots ,j$. Concretely, this means that *either(i)*$v=\theta +1$ and the rightmost $\theta +1$ positions in the interval [*i*, *j*] are all unpaired, *or(ii)* that $0 \le v < \theta +1$, and that position $j-v$ is paired to some $r \in [i,j-v-\theta -1]$. In base case D and inductive case D below, we will treat the two possible subcases of *(i)*, in which the rightmost $\theta +1$ positions are unpaired – namely, the subcase *(i)*$_a$ in which $j-\theta$ is unpaired (hence the rightmost $j-\theta +1$ positions are unpaired), and the subcase *(i)*$_b$ in which position $j-\theta$ is paired with some $r \in [i,j-v-\theta -1]$.

Let $Z^*(i,j,k)$ denote the number of secondary structures on the interval [*i*, *j*] that have exactly *k* neighbors with respect to the $MS_1$ move set (i.e. have degree *k*), so that

4 $$\begin{aligned} Z^*(i,j,k) = \sum _{h=0}^{j-\theta -i} \sum _{v=0}^{\theta +1} Z(i,j,k,h,v) \end{aligned}$$

Recalling from Eq. (1) that $\text{ maxDegree }(n) = \frac{(n-\theta )(n-\theta -1)}{2}$, for any $1 \le i\le j \le n$, we clearly have that

$$\begin{aligned} N(i,j)&= \sum _{k=1}^{\mathrm{maxDegree(j-i+1)}} Z^*(i,j,k) \\&= \sum _{k=1}^{\mathrm{maxDegree(j-i+1)}} \sum _{h=0}^{j-\theta -i} \sum _{v=0}^{\theta +1} Z(i,j,k,h,v) \end{aligned}$$

In the sequel, we describe Base Cases A–D, which initialize the arrays *Z*(*i*, *j*, *k*, *h*, *v*) and $Z^*(i,j,k)$, followed by Inductive Cases A–D, which treat the corresponding updates within the for-loops of the following pseudocode. Since arrays $Z,Z^*$ are initially set to zero, all updates to the arrays will be performed by adding a value val to the current value held in the array, so we will write $Z(i,j,k,h,v) \mathrel {+}=\text{ val }$ and $Z^*(i,j,k) \mathrel {+}=\text{ val }$, which is a standard abbreviation for the assignments $Z(i,j,k,h,v) = Z(i,j,k,h,v)+\text{ val }$ and $Z^*(i,j,k) = Z^*(i,j,k)+\text{ val }$. Explatory comments in the pseudocode are indicated by a double-slash. In Algorithm 1, assume a positive integer input of *n* to indicate a length *n* homopolymer.
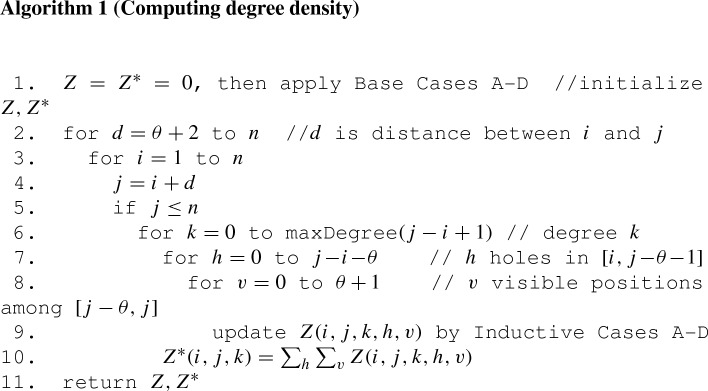


In line 1, arrays $Z,Z^*$ are initialized to zero, then Base Cases A–D are applied; lines 2–9 then treat the Inductive Cases A–D. In this dynamic programming (DP) algorithm, the idea is to define $Z,Z^*$ for all intervals [*i*, *j*] where $d=j-i$, after having computed and stored the values for $Z,Z^*$ for all intervals [*i*, *j*] where $j-i=d-1$. All secondary structures of the interval [*i*, *j*] can be partitioned into structures having exactly degree *k* (i.e. *k*$MS_1$ neighbors, in which *k* structures that can be obtained by either adding or removing a single base pair). To support an inductive argument, in proceeding from interval [*i*, *j*] to $[i,j+1]$, we need additionally to determine the number of structures having degree *k*, which have a certain number *h* of positions that are *visible* (external to every base pair), which can be paired with the last position $j+1$. Note that the position $j-\theta$ can *not* be base-paired with *j* in [*i*, *j*]; however, $j-\theta$*can* be base-paired with *j* in $[i,j+1]$. Thus in addition to keeping track of the number *h* of *holes* (positions in $i,\ldots ,j-\theta -1$ that are external to all base pairs, hence can be paired with *j*), we introduce the variable *v* to keep track of the number of *visible* positions in $j-\theta ,\ldots ,j$. This explains our need for the function *Z*(*i*, *j*, *k*, *h*, *v*) as defined in the Eqs. (5–12) for Base Cases A–D. We now proceed to the details, where for ease of the reader, some definitions are repeated.

Let $\theta =3$ denote the minimum number of unpaired positions required to be present in a hairpin loop. For a length *n* homopolymer, let $1 \le i \le j \le n$, $0 \le k \le {{n-\theta } \atopwithdelims ()2}$, $0 \le h \le j-i-\theta$, $0 \le v \le \theta +1$. Recall that *Z*(*i*, *j*, *k*, *h*, *v*) denotes the number of secondary structures on the interval [*i*, *j*], for $1 \le i \le j \le n$ for the homopolymer model, that have exactly *k* neighbors, and for which there are exactly *h* unpaired positions (or *holes*) in $[i,j-\theta -1]$, and for which there are exactly $v \in [0,\theta +1]$ visible positions among $j-\theta ,j-\theta +1,\ldots ,j$. For $0 \le v \le \theta$, this means that position $j-v$ is base-paired to some $r \in [i,j-v-\theta -1]$ while positions $j-v,j-v+1,\ldots ,j$ are not base-paired to any position in [*i*, *j*]. When $v=\theta +1$, this means simply that the rightmost $\theta +1$ positions in the interval [*i*, *j*] are all unpaired.

Recall as well our definition that $Z^*(i,j,k) = \sum _h \sum _v Z(i,j,k,h,v)$. We begin by initializing $Z(i,j,k,h,v)=0$ for all values in corresponding ranges. Letting *N*(*i*, *j*) denote the number of secondary structures on [*i*, *j*] for the homopolymer model, as computed by Eq. (2), the following recurrences describe an algorithm that requires $O(K \cdot n^3)$ storage and $O(K^2 \cdot n^4)$ time to compute the probability $Prob[ \mathrm{deg}(s) = k] = \frac{Z^*(1,n,k)}{N(1,n)}$ that a (uniformly chosen) random secondary structure has degree *k* for $0 \le k \le K$, where *K* is a user-defined constant bounded above by $\text{ maxDegree }(n) = \frac{(n-\theta )(n-\theta -1)}{2}$.

**Fig. 1:**
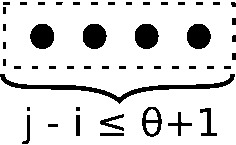
Structures considered in base case A

Base Case A considers all structures on [*i*, *j*], as depicted in Fig. 1, that are too small to have any base pairs, hence which have degree zero.

**Base Case A:** For $j-i \le \theta$, define

5 $$\begin{aligned} Z(i,j,0,0,j-i+1)&= 1 \end{aligned}$$

**Fig. 2:**
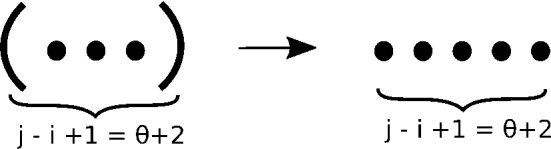
Structures considered in base case B

Base Case B considers all structures on [*i*, *j*], as depicted in Fig. 2, that have only base pair (*i*, *j*), since other potential base pairs would contain fewer than $\theta$ unpaired bases. The degree of such structures is 1, since only one base pair can be removed, and no base pairs can be added. Moreover, no position in [*i*, *j*] is external to the base pair (*i*, *j*), so visibility parameters $h=0,v=0$. The arrow in Fig. 2 indicates that the sole neighbor is the empty structure, obtained by removing the base pair (*i*, *j*).

**Base Case B:** For $j-i = \theta +1$ and (*i*, *j*) is a base pair, define

6 $$\begin{aligned} Z(i,j,1,0,0)&=1 \end{aligned}$$

**Fig. 3:**
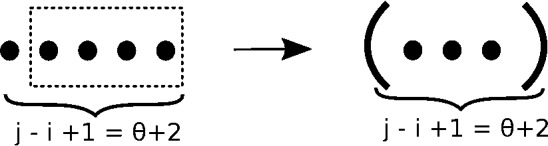
Structures considered in base case C

Base Case C considers the converse situation, consisting of the empty structure on [*i*, *j*] where $j-i = \theta +1$, whose sole neighbor is the structure consisting of base pair (*i*, *j*). The arrow is meant to indicate that the structure on the right is the only neighbor of that on the left, as depicted in Fig. 3. Since the size of the empty structure on [*i*, *j*] is $\theta +2$ and every position in [*i*, *j*] is visible (external to every base pair), $h=1$ and $v=\theta +1$. the dotted rectangle in Fig. 3 indicates the $\theta +1$ unpaired positions at the right extremity as counted by $v=\theta +1$.

**Base Case C:** For $j-i = \theta +1$ and (*i*, *j*) not base-paired, define

7 $$\begin{aligned} Z(i,j,1,1,\theta +1)&=1 \end{aligned}$$

**Fig. 4:**
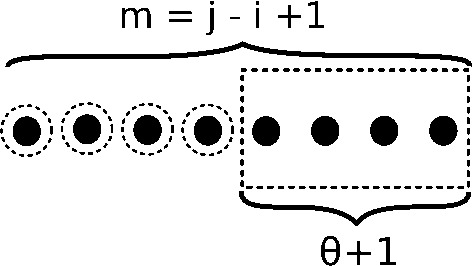
Structures considered in base case D

Base Case D considers the empty structure on [*i*, *j*] where $j-i>\theta +1$. The empty structure is the only structure having degree maxDegree$(i,j) = \frac{(j-i-\theta +1)(j-i-\theta )}{2}$, since maxDegree(*i*, *j*) many base pairs can be added to the empty structure. In Fig. 4, the dotted rectangle indicates the $\theta +1$ rightmost unpaired positions, corresponding to visibility parameter $v=\theta +1$, while dotted circles indicate the $h = j-i-\theta$ holes, i.e. unpaired positions that could be paired with the rightmost position *j*.

**Base Case D:** For all $(j-i+1) > \theta +2$, the empty structure, as indicated by $h+v=j-i+1$ (so $h=j-i-\theta$ and $v=\theta +1$), has degree maxDegree(*i*, *j*) as defined by Eq. 1, where

8 $$\begin{aligned} Z(i,j,\text{ maxDegree }(i,j),j-i-\theta ,\theta +1)&=1 \end{aligned}$$

Inductive Case A considers the case where left and right extremities *i*, *j* form the base pair (*i*, *j*), where $j-i>\theta +1$. No position in [*i*, *j*] is visible (external to all base pairs), so visibility parameters $h=0=v$. Recalling the definition of $Z^*(i,j,k)$ from Eq. 4, we have the following.

**Inductive Case A:** For $j-i > \theta +1$ and (*i*, *j*) base-paired in [*i*, *j*],

9 $$\begin{aligned} Z(i,j,k,0,0)&= Z(i,j,k,0,0) + Z^*(i+1,j-1,k-1) \end{aligned}$$

**Fig. 5:**
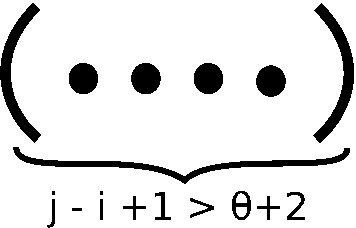
Structures considered in inductive case A

From this point on, we use the operator $\mathrel {+}=$, so that the previous equation would be written as $Z(i,j,k,0,0) \mathrel {+}=Z^*(i+1,j-1,k-1)$ (see Fig. 5).

**Fig. 6:**
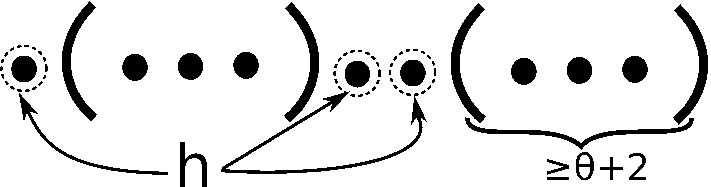
Structures considered in inductive case B

Inductive Case B considers the case where last position *j* base-pairs with the *r*, where $i<r<j-\theta$. The value $r=i$ has already been considered in Inductive Case A, and values $r=j-\theta +1,\ldots ,j-1$ cannot base-pair to *j*, since the corresponding hairpin loop would constain less than $\theta$ unpaired positions. This situation is depicted in Fig. 6, where there are *h* holes (positions in $[i,j-\theta -1]$ that are external to all base pairs) and no visible positions in $[j-\theta ,j]$.

**Inductive Case B:** For $j-i > \theta +1$ and (*r*, *j*) base-paired in [*i*, *j*] for some $i<r<j-\theta$,

10 $$\begin{aligned}&Z(i,j,k,h,0) \nonumber \\&\quad += \sum \limits _{r=i+1}^{j-\theta -1} \sum \limits _{k_1+k_2 = k-1} \sum \limits _{w=0}^{\theta +1} Z(i,r-1,k_1,h-w,w) \cdot Z^*(r+1,j-1,k_2) \end{aligned}$$

When implemented, this requires a check that $h-w \ge 0$.

**Fig. 7:**
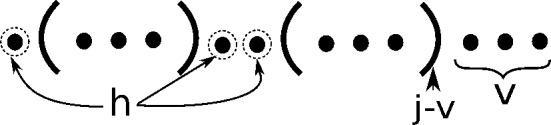
Structures considered in inductive case C(*v*)

For each value $v \in \{1,\ldots ,\theta +1\}$, inductive Case *C*(*v*) considers the case where position $r \in [i,j-v-\theta -1]$ forms a base pair with position $j-v$. The value $v=0$ is not considered here, since it was already considered in Inductive Cases A,B. Note that a structure *s* of the format has *k* neighbors, provided the restriction of *s* to $[i,r-1]$ has $k_1$ neighbors, and the restriction of *s* to $[r+1,j-1]$ has $k_2$ neighbors, where $k_1+k_2+vh+1=k$. The term *vh* is due to the fact that since base pair $(r,j-v)$ ensures that all *holes* are located in $[i,r-1]$, hence located at more than $\theta +1$ distance from all *visible* positions in $[j-v+1,j]$, a neighbor of *s* can be obtained by adding a base pair from any hole to any visible suffix position—there are *vh* many such possible base pairs that can be added. Finally, the last term $+1$ is present, since one neighbor of *s* can obtained by removing base pair $(r,j-v)$. This explains the summation indices and summation terms in Eq. (11). Figure 7 depicts a typical structure considered in case *C*(*v*).

**Inductive Case C**(*v*), **for**$v \in \{1,2,\ldots ,\theta +1\}$: For $j-i > \theta +1$ and $(r,j-v)$ base-paired in [*i*, *j*], for some $i<r<j-v-\theta$, where $j-v+1,\ldots ,j$ are unpaired in [*i*, *j*],

11 $$\begin{aligned} Z(i,j,k,h,v)&+= Z^*(2,j-1-v,k-1-vh) \nonumber \\&+ \sum \limits _{r=i+1}^{j-v-\theta -1} \sum \limits _{k_1+k_2=(k-1-v h)} \nonumber \\&\qquad \sum \limits _{w=0}^{\theta +1} Z(i,r-1,k_1,h-w,w) \cdot Z^*(r+1,j-1-v,k_2) \end{aligned}$$

The first term $Z^*(2,j-1-v,k-1-vh)$ handles the subcase where $r=1$, so that $(1,j-v)$ is a base pair, while the second term handles the subcase where $r>1$. Note that when implemented, this requires a test that $h-w\ge 0$.

Case *D* considers the case where there are *h* holes, and positions $j-\theta -1,\ldots ,j$ are unpaired, so that $v=\theta +1$. Note that $v=\theta +1$ implies only that $j-\theta ,\ldots ,j$ are unpaired, so Case *D* includes the addition requirement that position $j-\theta -1$ is unpaired. Structures *s* satisfying Case *D* can be partitioned into subcases where the restriction of *s* to $[i,j-\theta -1]$ has $h-w$ holes in $[i,(j-\theta -1)-(\theta +1)] = [i,j-2\theta -2]$, and $1 \le w \le \theta +1$ visible positions in $[j-2\theta -1,j-\theta -1]$. Note that $(h-w)+w=h$, accounting for the *h* holes in structure *s* in $[i,j-\theta -1]$, and that it is essential that $w\ge 1$, since the case $w=0$ was considered in Case $C(\theta +1)$.

The term $\frac{w(w+1)}{2}$ is due to the fact that the rightmost position $j-\theta -1$ in the restriction of *s* to $[i,j-\theta -1]$ can base-pair with position *j*, but not with $j-1$, etc. since this would violate the requirement of at least $\theta$ unpaired bases in a hairpin loop. Similarly, the second rightmost position $j-\theta -2$ in the restriction of *s* to $[i,j-\theta -1]$ can base-pair with positions *j* and $j-1$, but not with $j-2$, etc.; as well, the third rightmost position $j-\theta -3$ can base-pair with positions *j*, $j-1$ and $j-2$, but not with $j-3$, etc. The number of neighbors of *s* produced in this fashion is thus $\sum _{i=1}^w i = \frac{w(w+1)}{2}$. Finally, the term $(\theta +1)(h-w)$ is due to the fact that each of the $h-w$ holes in the restriction of *s* to $[i,j-\theta -1]$ can base-pair to each of the $(\theta +1)$ positions in $[j-\theta ,j]$.

The argument just given shows the following. Let *s* be a structure that satisfies conditions of Case *D* with *h* holes and $v=\theta +1$ visible positions, and suppose that the restriction of *s* to $[i,j-\theta -1]$ has $h-w$ holes and *w* visible positions. Then *s* has *k* neighbors provided that the restriction of *s* to $[i,j-\theta -1]$ has $k-\frac{w(w+1)}{2} - (\theta +1)(h-w)$ neighbors on interval $[i,j-\theta -1]$. The Eq. (12) now follows.

**Inductive Case D:** For $j-i > \theta +1$ and $j-\theta -1,j-\theta ,\ldots ,j$ unpaired in [*i*, *j*], and $1 \le h < j-\theta -i$,

12 $$\begin{aligned}&Z(i,j,k,h,\theta +1) \nonumber \\&\quad += \sum \limits _{w=1}^{\theta +1} Z(i,j-\theta -1,k- \frac{w(w+1)}{2} - (\theta +1)\cdot (h-w), h-w,w) \end{aligned}$$

**Fig. 8:**
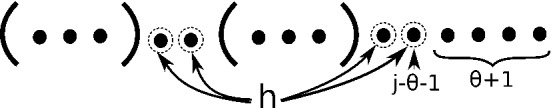
Structures considered in inductive case D

As in Case C(*v*), when implemented, this requires a test that $h-w\ge 0$ (see Fig. 8).

Our implementation of Eqs. (5–12) has been cross-checked with exhaustive enumeration; moreover, we always have that $\sum _{k} Z^*(i,j,k) = N(i,j)$, so the degree density is correctly computed.

### Faster algorithm in the homopolymer case

The algorithm described in Sect. 2.2 requires $O(K^2n^4)$ time and $O(Kn^3)$ space, where *K* is a user-specified degree bound $K \le \frac{(n-\theta )(n-\theta -1)}{2}$. By minor changes, that algorithm can be modified to compute the degree density function $p(k) = \frac{Z^*(1,n,k)}{N(1,n)}$ for any given RNA sequence $a_1,\ldots ,a_n$. In the case of a homopolymer, any two positions are allowed to base-pair (regardless of whether the base pair is a Watson–Crick or wobble pair), provided only that every hairpin loop contains at least $\theta$ unpaired positions. For homopolymers, we have a faster algorithm that requires $O(K^2 n^3)$ time and $O(Kn^2)$ space. Since nucleotide identity is unimportant, instead of *Z*(*i*, *j*, *k*, *h*, *v*), we describe the function ${{{\widehat{Z}}}} (m,k,h,v)$, where *m* corresponds to the length $j-i+1$ of interval [*i*, *j*].

$$\begin{aligned} {{{\widehat{Z}}}} ^*(m,k)&= \sum _{h=0}^{m-\theta -1} \sum _{v=0}^{\theta +1} {{{\widehat{Z}}}} (m,k,h,v) \\ N(m)&= \sum _{k=1}^{\frac{(m-\theta )(m-\theta -1)}{2}} {{{\widehat{Z}}}} ^*(m,k) \end{aligned}$$

We begin by initializing ${{{\widehat{Z}}}} (m,k,h,v)=0$ for all $1 \le m \le n$, $0 \le k \le \frac{(m-\theta )(m-\theta -1)}{2}$, $0 \le h \le m-2$, and $0 \le v \le \theta +1$. If $h<0$, we assume that ${{{\widehat{Z}}}} (m,k,h,v)=0$.

**Base Case A:** For $1 \le m \le \theta +1$, define

13 $$\begin{aligned} {{{\widehat{Z}}}} (m,0,0,m)&= 1 \end{aligned}$$

**Base Case B:** For $m = \theta +2$, define

14 $$\begin{aligned} {{{\widehat{Z}}}} (m,1,0,0)&=1 \end{aligned}$$

**Base Case C:** For $m = \theta +2$, define

15 $$\begin{aligned} {{{\widehat{Z}}}} (m,1,1,\theta +1)&=1 \end{aligned}$$

**Base Case D:** For all $m > \theta +2$, define

16 $$\begin{aligned} {{{\widehat{Z}}}} (m,\frac{(m-\theta )(m-\theta -1)}{2},m-\theta -1,\theta +1)&=1 \end{aligned}$$

**Inductive Case A:** For $m > \theta +2$ and $1 \le k \le \frac{(m-\theta )(m-\theta -1)}{2}$, define

17 $$\begin{aligned} {{{\widehat{Z}}}} (m,k,0,0)&\mathrel {+}={{{\widehat{Z}}}} ^*(m-2,k-1) \end{aligned}$$

**Inductive Case B:** For $m > \theta +2$, $1 \le k < \frac{(m-\theta )(m-\theta -1)}{2}$, and $0 \le h \le m-\theta -1$, define

18 $$\begin{aligned} {{{\widehat{Z}}}} (m,k,h,0)&\mathrel {+}=\sum \limits _{r=2}^{m-\theta -1} \sum \limits _{k_1+k_2 = k-1} \sum \limits _{w=0}^{\theta +1} {{{\widehat{Z}}}} (r-1,k_1,h-w,w) \cdot {{{\widehat{Z}}}} ^*(m-r-1,k_2) \end{aligned}$$

When implemented, this requires a check that $h-w \ge 0$.

**Inductive Case C**(*v*): For $v \in \{1,2,\ldots ,\theta +1\}$ and $m > \theta +2$, define

19 $$\begin{aligned} {{{\widehat{Z}}}} (m,k,h,v)&\mathrel {+}={{{\widehat{Z}}}} ^*(m-v-2,k-1-vh) \nonumber \\&+ \sum \limits _{r=2}^{m-v-\theta -1} \sum \limits _{k_1+k_2=(k-1-v h)} \nonumber \\&\qquad \sum \limits _{w=0}^{\theta +1} {{{\widehat{Z}}}} (r-1,k_1,h-w,w) \cdot {{{\widehat{Z}}}} ^*(m-v-r-1,k_2) \end{aligned}$$

**Inductive Case D:** For $m > \theta +2$, $1 \le k < \frac{(m-\theta )(m-\theta -1)}{2}$, and $1 \le h < m-\theta -1$,

20 $$\begin{aligned}&{{{\widehat{Z}}}} (m,k,h,\theta +1) \nonumber \\&\quad \mathrel {+}=\sum \limits _{w=1}^{\theta +1} {{{\widehat{Z}}}} (m-\theta -1,k- \frac{w(w+1)}{2} - (\theta +1)\cdot (h-w), h-w,w) \end{aligned}$$

Note that *h* is strictly less than $m-\theta -1$, since the case $h=m-\theta -1$ occurs only when additionally $v=\theta +1$, which only arises in the empty structure. The general case for the empty structure was handled in Base Case D. When implemented, this requires a check that $h-w \ge 0$.

## Statistical methods

Current software for probability distribution fitting of connectivity data, such as Matlab™, Mathematica™, R and powerlaw (Alstott et al. 2014), appear to require an input file containing the connectivity of each node in the network. In the case of RNA secondary structures, this is only possible for very small sequence length. To analyze connectivity data computed by the algorithm of Sect. 2.3, we had to implement code to compute the maximum likelihood estimation for scaling factor $\alpha$ in a power-law fit, the optimal degree $k_{min}$ beyond which connectivity data is fit by a power-law, and the associated *p* value for Kolmogorov–Smirnov goodness-of-fit, as described in Clauset et al. (2009). We call the resulting code RNApowerlaw. This section explains those details.

Recall the definition of the *zeta function*

21 $$\begin{aligned} \zeta (\alpha )&= \sum _{n = n_0}^{\infty } n^{-\alpha } \end{aligned}$$

We use both the generalized zeta function (22), as well as the truncated generalized zeta function (23), defined respectively by

22 $$\begin{aligned} \zeta (\alpha ;n_0)&= \sum _{n = n_0}^{\infty } n^{-\alpha } \end{aligned}$$

23 $$\begin{aligned} \zeta (\alpha ;n_0,n_1)&= \sum _{n = n_0}^{n_1} n^{-\alpha } \end{aligned}$$

Given a data set $D = \{x_1,\ldots ,x_n\}$ of positive integers in the range $[k_0,k_1]$, the likelihood $L(D|\alpha )$ that the data fits a truncated power-law with scaling factor $\alpha$ and range $[k_0,k_1]$ is defined by

24 $$\begin{aligned} L(D|\alpha )&= {\varPi }_{i=1}^n \frac{x_i^{-\alpha }}{\zeta (\alpha ;k_0,k_1)} \end{aligned}$$

Rather than sampling individual RNA secondary structures to estimate the connectivity of the secondary structure network for a given homopolymer, the algorithms from Sects. 2.2 and 2.3 directly compute the exact number *N*(*k*) of secondary structures having degree *k*, for all *k* within a certain range. It follows that the likelihood $L(D|\alpha )$ that secondary structure connectivity fits a power-law with scaling factor $\alpha$ is given by

25 $$\begin{aligned} L(D|\alpha ,k_0,k_1)&= {\varPi }_{k=k_0}^{k_1} \left( \frac{k^{-\alpha }}{\zeta (\alpha ;k_0,k_1)} \right) ^{N(k)} \end{aligned}$$

hence the log likelihood is is given by

26 $$\begin{aligned} {\mathcal {L}}(D|\alpha ,k_0,k_1)&= - \left( \log (\zeta (\alpha ;k_0,k_1)) \sum _{k=k_0}^{k_1} N(k) \right) - \left( \alpha \sum _{k=k_0}^{k_1} N(k) \log (k) \right) \end{aligned}$$

The parameter ${\widehat{\alpha }}$ which maximizes the log likelihood is determined by applying SciPy function minimize (with Nelder-Mead method) to the negative log likelihood, starting from initial estimate $\alpha _0$, taken from equation (3.7) of Clauset et al. (2009)

27 $$\begin{aligned} \alpha _0&= 1 + n \left( \sum _{i=1}^n \ln \frac{x_i}{x_{\mathrm{min}}- 1/2} \right) ^{-1} \end{aligned}$$

which in our notation yields

28 $$\begin{aligned} \alpha _0&= 1 + \left( \sum _{k=k_0}^{k_1} N(k) \right) \cdot \left\{ \sum _{k=k_0}^{k_1} N(k) \cdot \log \left( \frac{k}{k_0 - 1/2} \right) \right\} ^{-1} \end{aligned}$$

In results and tables of this paper, we often write the maximum likelihood estimate (MLE) ${\widehat{\alpha }}$ simply as $\alpha$.

We compute the Kolmogorov–Smirnov (KS) *p* value, following (Clauset et al. 2009), as follows. Given observed relative frequency distribution *D* and a power-law fit *P* with scaling factor $\alpha$, the KS distance is defined to be the maximum, taken over all $k \in [k_0,k_1]$ of the absolute difference between the cumulative distribution function (CDF) for the data evaluated at *k*, and the CDF for the power-law, evaluated at *k*

29 $$\begin{aligned} KS(k_{min},k_{max})&= \max _{k_{min} \le x \le k_{max}} |C_a(x) - C_f(x)| \end{aligned}$$

where $C_a$ and $C_f$ are the actual and fitted cumulative density functions, respectively. The KS *p* value for the fit of data *D* by power-law *P* with scaling factor $\alpha$, is determined by (1) sampling a large number ($N=1000$) of synthetic data sets $D_i$ from a true power-law distribution with scaling factor $\alpha$, (2) computing the KS distance between each synthetic data set $D_i$ and its power law fit with MLE scaling factor $\alpha _i$, (3) reporting the proportion of KS distances that exceed the KS distance between the original observed data set and its power-law fit with scaling factor $\alpha$.

Following (Clauset et al. 2009), $k_{\mathrm{min}}$ is chosen to be that degree $k_0$, such that the KS distance for the optimal power-law fit is smallest. In contrast, $k_{max}$ is always taken to be the maximum degree in the input data. Our computation of *p* value for goodness-of-fit follows Sect. 4.1 of Clauset et al. (2009), with the exception that we not generate any synthetic data for values $k< k_{max}$, since the MLE scaling factor $\alpha$ is determined for the (normalized) distribution of data values in the interval $[k_{min}, k_{max}]$, a convention followed in Alstott et al. (2014). We have implemented Python code to compute $\alpha _0$, $\alpha$, $k_{min}$, KS distance, *p* value, etc. as described above. In Sect. 4, we compare results of our code with that from powerlaw (Alstott et al. 2014) for very small homopolymers. Though our code does not do lognormal fits, this is performed by powerlaw, where the density function for the lognormal distribution with parameters $\mu ,\sigma$ is defined by

30 $$\begin{aligned} p(x)&= \frac{ \exp \left( -\frac{(log(x)-\mu )^2}{2 \sigma ^2} \right) }{x \cdot \sqrt{2 \pi \sigma ^2}} \end{aligned}$$

In computing the *p* value for power-law goodness-of-fit using Kolmogorov–Smirnov statistics, it is necessary to sample synthetic data from a (discrete) power-law distribution with scaling factor $\alpha$, a particular type of multinomial distribution. Given an arbitrary multinomial distribution with probability $p_i$ for each $1\le i \le m$, it is straightforward to create *M* synthetic data sets, each containing *N* sampled values, in time *O*(*mNM*); however, since $M=1000$ and *N* is the (exponentially large) number of all secondary structures having degrees in $[k_{min}, k_{max}]$, the usual *sequential* method would require prohibitive run time. Instead, we implemented the much faster *conditional* method (Malefaki and Iliopoulos 2007). Our goal is to sample from a multinomial distribution given by

31 $$\begin{aligned} \displaystyle Prob\left[ X_1=x_1,X_2=x_2,\ldots ,X_{m} \right]&= \frac{N!}{\displaystyle \prod \nolimits _{i=1}^{m} x_i!} \displaystyle \prod _{i=1}^{m} p_i^{x_i} \end{aligned}$$

where $m = k_{max}-k_{min}+1$ is the number of degrees in the synthetic data, and in the sample set of size *N* there are $x_i$ many occurrences of degree $k_{min}+i$. To do this, we sample $X_1$ from the binomial distribution of *N* coin tosses with heads probability $p_1$, then $X_2$ from the binomial distribution of $N-x_1$ coin tosses with heads probability $\frac{p_2}{1-p_1}$, then $X_3$ from the binomial distribution of $N-x_1-x_2$ coin tosses with heads probability $\frac{p_2}{1-p_1-p_2}$, etc. where each $x_i$ is determined with the function binom from Python Scipy.stats.

**Table 1:** Table comparing goodness-of-fit computations for software powerlaw (Alstott et al. 2014) and RNApowerlaw for homopolymer lengths less than 30 nt

*n*	$S_n$	$k_{min}$	$\alpha$ (PL)	$\alpha$ (RNAPL)	KSdist (PL)	KSdist (RNAPL)	$\langle \text{ KSdist } \rangle$	log odds ratio R (PL)	*p*-val for R (PL)	*p*-val (RNAPL)
10	65	3	3.13752	3.13753	0.05576	0.05576	0.02721	$-$0.15	0.765	0.813
12	274	4	3.23011	3.23011	0.03650	0.03650	0.01277	$-$0.81	0.482	0.746
14	1184	5	3.38933	3.38935	0.02021	0.02021	0.00669	$-$1.70	0.270	0.699
16	5223	6	3.51285	3.51289	0.02252	0.02253	0.00603	$-$6.78	0.029	0.051
18	23,434	9	3.79069	3.79073	0.02333	0.02333	0.00624	$-$16.00	0.001	0.001
20	106,633	10	3.87168	3.87165	0.02116	0.02116	0.00581	$-$82.12	0.000	0.000
22	490,999	10	3.85806	3.85809	0.02304	0.02304	0.00523	$-$670.64	0.000	0.000
24	2,283,701	14	4.16480	4.16477	0.02242	0.02242	0.00484	$-$1452.24	0.000	0.000
26	10,713,941	15	4.24485	4.24486	0.02298	0.02298	0.00417	$-$7129.42	0.000	0.000
28	50,642,017	16	4.33086	4.33089	0.02167	0.02168	0.00347	$-$33,020.89	0.000	0.000
30	240,944,076	–	–	4.33681	–	0.02393	0.00298	–	–	0.000

Given homopolymer length *n*, the connectivity density is computed over all secondary structures for (all possible) degrees $k=1,\ldots ,\frac{(n-3)(n-4)}{2}$ using the algorithm described in Sect. 2.3. Program powerlaw requires an input file containing the degrees of all structures (i.e. containing $S_n$ values, where $S_n$ is the exponentially large number of all secondary structures), while our program RNApowerlaw requires as input a list of degrees and their (absolute) frequencies. Table headers as follows: *n* is homopolymer length, $S_n$ is the number of all secondary structures, $\alpha$ is the maximum likelihood value for the scaling factor of the optimal power-law fit, as computed by powerlaw (PL) and RNApowerlaw (RNAPL), KSdist is the Kolmogorov–Smirnov (KS) distance using Eq. (29), $\langle \text{ KSdist } \rangle$ is the mean KS-distance obtained by replacing ‘max’ by ‘mean’ in Eq. (29), *R* is the log-odds ratio with associated *p* value as computed by powerlaw, and the *p* value in the last column is computed by RNApowerlaw as described in Sect. 3. Since powerlaw required more than 24 h for the computation when $n=28$, we did not attempt a computation for $n=30$; in contrast, RNApowerlaw requires a few seconds computation time. Since the log-odds ratio *R* is the logarithm of the power-law likelihood divided by lognormal likelihood, a negative value $R<0$ indicates that the lognormal distribution is a better fit for the tail of RNA secondary structure connectivity data. A small *p* value computed by RNApowerlaw indicates that RNA connectivity data is not well-approximated by a power-law distribution. While our code RNApowerlaw computes the *p* value for the power-law fit, Alstott’s code powerlaw only computes the *p* value for the log-odds ratio test

## Results

Below, we use the algorithms described in previous sections to compute RNA secondary structure connectivity, determine optimal scaling factor $\alpha$ and minimum degree $k_{min}$ for a power-law fit, then perform Kolmogorov–Smirnov bootstrapping to determine the goodness-of-fit for parameters $\alpha ,k_{min}$. In Appendix A, we show that preferential attachment appears to hold for the network of RNA structures, at least for our definition of preferential attachment.

### Analysis of RNA networks using RNAdensity and RNApowerlaw

**Fig. 9:**
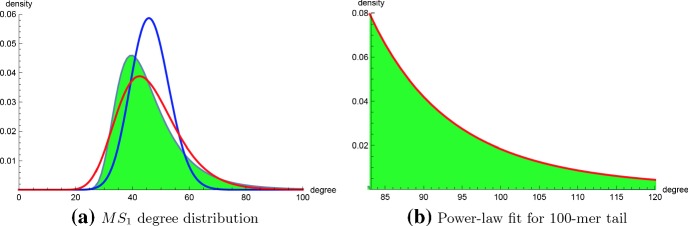
**a** Connectivity degree distribution for homopolymer of length 100 where $\theta =3$, computed with the algorithm described in Sect. 2.3 for all degrees bounded by $K = 200$. There are $6.32 \cdot 10^{32}$ secondary structures for the 100-mer (exact number 6.31986335936396855341222902079183), and $99.9978706904\%$ of the structures have degree bounded by *K*. Using the output degree densities, the degree mean [standard deviation] is $\mu =46.2543801196$ [resp. $\sigma =12.2262985078$]; note that the mean computed from the algorithm in Sect. 2.3 is very close to the exact degree mean of $\mu =46.2591895818$, computed over all structures using the different dynamic programming algorithm in Clote (2015). The Poisson distribution (blue curve) with same mean $\mu$ is shown, as well as the lognormal distribution (red) with parameters $\mu _0=3.80467214577$ and $\sigma _0=0.235563374146$; i.e. $\mu _0$ [resp. $\sigma _0$] is the mean [resp. standard deviation] for logarithms of the connectivity degree—see Eq. (30). **b** Power-law fit of tail with scaling factor $\alpha =7.8762287746$ and $k_{min}=83$, determined by maximum likelihood. Kolmogorov–Smirnov (KS) distance for the fit is 0.01213—see Eq. (29), while average KS distance for the alpha power-law fit 0.00400. Nevertheless, since the *p* value 0 (to 10 decimal places), hypothesis testing would reject the null hypothesis that the power-law distribution is a good fit for the tail (color figure online)

The algorithm RNAdensity described in Sect. 2.3 was used to compute absolute and relative degree frequencies for the following cases: (1) homopolymers of length $n=10,12,\ldots ,40$ with $\theta =3$ for maximum possible degree upper bound $K= \frac{(n-\theta )(n-\theta -1)}{2}$, (2) homopolymers of length $n=30,35,\ldots ,150$ with $\theta =3$, where degree upper bound $K=2n$ for $n\in [30,100]$ and $K=n+30$ for $n \in [105,150]$, (3) homopolymers of length $n=30,35,\ldots ,150$ with $\theta =1$, where degree upper bound $K=2n$ for $n\in [30,100]$ and $K=n+30$ for $n \in [105,150]$. For small homopolymers of length at most 30, optima values for $k_{min}$, power-law scaling factor $\alpha$, Kolmogorov–Smirnov distance were determined using software powerlawpowerlaw (Alstott et al. 2014) as well as RNApowerlaw from Sect. 3. Table 1 summarizes these results, which show the agreement between powerlaw and RNApowerlaw. Moreover, both both programs indicate that formal hypothesis testing rejects the null hypothesis that a power-law distribution fits connectivity data; indeed, powerlaw determines a negative log odds ratio *R* for the logarithm of power-law likelihood over lognormal likelihood, indicating a better fit for the lognormal distribution, and RNApowerlaw determines small *p* values for Kolmogorov–Smirnov goodness-of-fit of a power-law distribution. Figure 9a shows connectivity density function for a 100-mer, with overlaid Poisson and lognormal distributions—since Erdös-Rényi random graphs have a Poisson degree distribution (Albert and Barabási 2002), it follows that RNA secondary structure networks are strikingly different than random graphs. Figure 9b shows a portion of the power-law fit for degrees in $[k_{min}, k_{max}]$, where scaling factor $\alpha \approx 7.876$ and $k_{min}=83$. Although maximum degree probability at $k_{{peak}}$ is less than 0.05 for the raw data, the connectivity density for $[k_{min}, k_{max}]$ is normalized, which explains why the degree probability for $k_{min}$ is $\approx 0.08$. Visual inspection might suggest an excellent fit for the power-law distribution; however, a Kolmogorov–Smirnov *p* value of $\approx 0$ indicates that the distribution is not power-law. The seemingly good power-law fit for RNA connectivity data, suggested by visual inspection, however motivated our initial investigation of preferential attachment.

**Table 2:** Table showing that approximate [resp. exact] scaling factor $\alpha _0$ [resp. $\alpha$] and minimum degree $k_{min}$ for optimal power-law fit of homopolymer connectivity data can not be reliably computed by using software powerlaw (Alstott et al. 2014) on data sampled from relative frequencies

*N*	$10^2$	$10^3$	$10^4$	$10^5$	$10^6$	$10^7$	RNAPL	$S_n$
$\alpha _0$, $n=20$	6.58318	3.66505	3.93389	3.86017	3.84749	3.84657	3.84648	$106633\approx 1.1 \cdot 10^5$
$k_{min}$	10	7	10	10	10	10	10	–
$\alpha _0$, $n=30$	5.27581	4.42183	4.46307	4.35008	4.32651	4.32272	4.32213	$240944076 \approx 2.4 \cdot 10^{8}$
$k_{min}$	12	13	16	16	16	16	16	–
$\alpha _0$, $n=40$	5.15978	5.09714	5.03719	5.24488	5.16985	5.70916	5.94561	$633180247373\approx 6.3 \cdot 10^{11}$
$k_{min}$	15	19	23	29	29	42	49	–
$\alpha$, $n=20$	6.76575	3.70988	3.96139	3.88570	3.87271	3.87180	3.87165	$106633\approx 1.1 \cdot 10^5$
$k_{min}$	10	7	10	10	10	10	10	–
$\alpha$, $n=30$	5.33162	4.44651	4.47963	4.36511	4.34122	4.33739	4.33681	$240944076 \approx 2.4 \cdot 10^{8}$
$k_{min}$	12	13	16	16	16	16	16	–
$\alpha$, $n=40$	5.19197	5.11604	5.049419	5.25365	5.17824	5.65206	5.95033	$633180247373\approx 6.3 \cdot 10^{11}$
$k_{min}$	15	19	23	29	29	41	49	–

Approximate value $\alpha _0$ is computed from Eq. (27), while $\alpha$ is the maximum likelihood estimate (MLE) of the optimal power-law scaling factor. Given homopolymer length $n=20,30,40$, connectivity density is computed over all secondary structures for (all possible) degrees $k=1,\ldots ,\frac{(n-3)(n-4)}{2}$ using the algorithm described in Sect. 2.3. Since powerlaw requires input files of (individually observed) connectivity degrees, rather than a histogram of (absolute) frequencies *F*(*k*) of connectivity degrees, we generated a file consisting of $N \cdot p(k)$ many occurrences of the value *k*, where $N=10^2,10^3,\ldots ,10^7$ denotes the total number of samples, and where relative frequency *p*(*k*) is defined by $p(k) = F(k)/\sum _{k=1}^{(n-3)(n-4)/2} F(k)$. In contrast to powerlaw, our program RNApowerlaw (RNAPL) computes *exact* values from connectivity degree (absolute) frequencies. When using powerlaw, it is clearly necessary to create input files of ever-increasing sizes *N*, in order to have more accurate values of $\alpha _0$, $\alpha$ and $k_{min}$. Since the number $S_n$ of RNA secondary structures is exponential in homopolymer length *n*, it rapidly becomes impossible to use powerlaw for large RNAs—for instance, table values for $n=40$ required an overnight run of powerlaw, while our software returned the exact value within a few seconds

**Table 3:** Table showing maximum likelihood scaling factors $\alpha$ with associated *p* values for optimal power-law fits of RNA secondary structure connectivity data for homopolymers of length $n=30$ to 150

*n*	$k_{max}$	$\%$ of $S_n$	$k_{{peak}}$	$k_{{mfe}}$	$k_{min}$	$\alpha (k_{min}, k_{max})$	$KS (k_{min}, k_{max})$	$\langle KS \rangle$	*p* val
30	60	0.998861	10	13	16	4.223674	0.014393	0.004877	0.000000
35	70	0.999174	12	16	18	4.395936	0.015391	0.005284	0.000000
40	80	0.999404	14	18	23	4.736679	0.015298	0.004859	0.000000
45	90	0.999563	16	21	30	5.146670	0.012075	0.004291	0.000000
50	100	0.999681	18	23	32	5.310801	0.012421	0.004345	0.000000
55	110	0.999762	20	26	39	5.674231	0.011649	0.003979	0.000000
60	120	0.999823	22	28	41	5.829310	0.012328	0.003979	0.000000
65	130	0.999866	24	31	49	6.200720	0.010772	0.003572	0.000000
70	140	0.999899	26	33	52	6.386452	0.010836	0.003464	0.000000
75	150	0.999923	28	36	60	6.721588	0.009719	0.003151	0.000000
80	160	0.999941	31	38	63	6.897103	0.009818	0.003067	0.000000
85	170	0.999955	33	41	67	7.097544	0.009737	0.002940	0.000000
90	180	0.999965	35	43	74	7.373569	0.008916	0.002726	0.000000
95	190	0.999973	37	46	78	7.564208	0.008755	0.002615	0.000000
100	200	0.999979	40	48	83	7.775022	0.008444	0.002476	0.000000
105	135	0.999388	42	51	67	7.204937	0.010712	0.003853	0.000000
110	140	0.999432	44	53	70	7.360192	0.010810	0.003854	0.000000
115	145	0.999474	46	56	73	7.513728	0.010889	0.003852	0.000000
120	150	0.999512	49	58	77	7.706717	0.010405	0.003703	0.000000
125	155	0.999549	51	61	80	7.856962	0.010504	0.003696	0.000000
130	160	0.999582	53	63	84	8.045458	0.010102	0.003556	0.000000
135	165	0.999614	55	66	88	8.231267	0.009724	0.003425	0.000000
140	170	0.999643	58	70	91	8.377410	0.009809	0.003418	0.000000
145	175	0.999670	60	71	94	8.522170	0.009884	0.003413	0.000000
150	180	0.999695	62	75	98	8.703041	0.009515	0.003289	0.000000

Absolute and relative connectivity degree frequencies were computed by RNAdensity from Sect. 2.3, while the optimal parameters $\alpha , k_{min}$ and *p* values were computed by RNApowerlaw from Sect. 3. Column headers are as follows: *n* is sequence length, $k_{max}$ is the degree upper bound *K* for RNAdensity, $\%$ of $S_n$ indicates the proportion of all secondary structures having degree bounded by $K=k_{max}$, $k_{{peak}}$ is the location of the density maximum, $k_{{mfe}}=\lfloor \frac{n-\theta }{2} \rfloor$ is the degree of the minimum free energy structure (having largest number of base pairs), $k_{min}$ is the optimal lower bound for a power-law fit, $\alpha (k_{min},k_{max})$ is the maximum likelihood scaling factor for power-law fit, $KS(k_{min},k_{max})$ is the Kolmogorov–Smirnov (KS) distance between connectivity data and power-law fit, *p* val is goodness-of-fit *p* value for Kolmogorov–Smirnov statistics, and $\langle KS \rangle$ is the average KS distance, obtained by replacing ‘max’ by ‘mean’ in Eq. (29)

Since powerlaw requires input files of (individually observed) connectivity degrees, when creating Table 1, we could not run powerlaw for homopolymer length greater than 28, for which latter the input file contained 50, 642, 017 values. A potentially attractive alternative is to generate input files consisting of $N \cdot p(k)$ many occurrences of the value *k*, where $N=10^2,10^3,\ldots ,10^7$ denotes the total number of samples, and where relative frequency *p*(*k*) is the proportion of structures having degree *k*. However, Table 2 shows that neither scaling factor $\alpha$ nor $k_{min}$ are correct with this alternative approach, even for small homopolymers of length 20, 30 and 40. This table justifies the need for our implementation of RNApowerlaw as described in Sect. 3. Table 3 shows maximum likelihood scaling factors $\alpha$ and Kolmogorov–Smirnov *p* values for optimal power-law fis of connectivity data for homopolymers of lengths from 30 to 150.

**Fig. 10:**
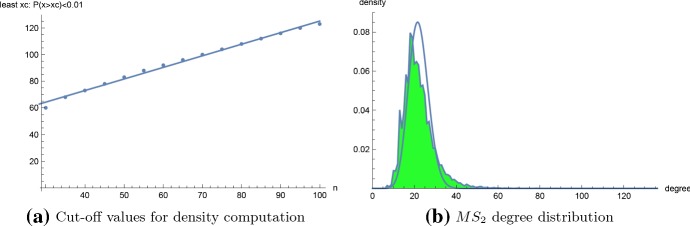
**a** Plot of the least cut-off value $x_c$ as a function of homopolymer length *n*, for $n=30,40,\ldots ,100$. Here $x_c$ is defined as the least value such that the probability that a secondary structure for length *n* homopolymer has degree greater that $x_c$ is at most 0.01. For the least-squares fit, the regression equation is $y=0.870714 x + 38.1369$, with *p* value of $1.65112 \cdot 10^{-15}$ for slope value, and *p* value of $5.20963 \cdot 10^{-13}$ for the *y*-intercept. **b**$MS_2$ connectivity for the 106,633 secondary structures for a 20-nt homopolymer with $\theta =3$ (green shaded curve), with Poisson distribution of the same mean. Connectivity values range from $4,\ldots ,136$ (with many intermediate gaps before the max degree). The distribution mean [resp. standard deviation] is $\mu =22.0531$ [resp. $\sigma =7.333$]; these values should be contrasted with the corresponding values of $\mu '=8.3364$ [resp. $\sigma '=4.7690$] for $MS_1$ connectivity for the same 20-nt homopolymer (data not shown) (color figure online)

**Fig. 11:**
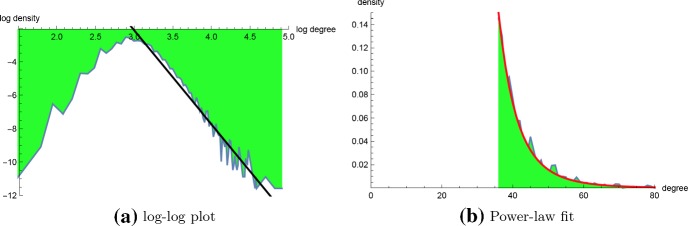
**a** Plot of $\ln (\text{ density })$ as a function of $\ln (\text{ degree })$ for the degree distribution for $MS_2$ connectivity of the 20-nt homopolymer with $\theta =3$, for degrees $4,\ldots ,136$. The distribution tail appears to satisfy a power-law with exponent $\approx -5.6247$, i.e. $p(x) \propto x^{-5.6247}$, where *x* is degree and *p*(*x*) is the relative frequency of the number of nodes having degree *x* (regression equation log-log plot is $\ln (p(x)) = 14.7589 - 5.6247 \cdot x$). **b** It is well-known that linear regression of the log-log plot is less reliable than using maximum likelihood when establishing whether the tail of empirical data is fit by a power-law distribution. For the $MS_2$ connectivity data of a 20-nt homopolymer, the maximum likelihood estimation (MLE) of optimal power-law scaling factor is $\alpha =6.8257$ with *p* value is 0.219 when $k_{min}=36$ and $k_{max}=136$. Since the *p* value is not less than 0.05, we can not reject the null hypothesis that $MS_2$ connectivity is well-fit by a power-law distribution (color figure online)

Figure 10a shows a scatter plot with regression line for the *cut-off* values $x_c$, defined to be the least value such that the probability that a secondary structure for length *n* homopolymer has degree greater that $x_c$ is at most 0.01. From this figure, we determined that for homopolymer length $n>100$, it more than suffices to take degree upper bound $K=n+30$. Figure 10b shows the connectivity degree distribution for a homopolymer of length 20, where degree *dg*(*s*) is redefined to be the number of structures *t* that can be obtained from *s* by adding, removing, or *shifting* a base pair in *s*. The so-called $MS_2$ move set, consisting of an addition, removal or shift of a base pair is the default move set used in RNA kinetics software kinfold (Lorenz et al. 2011). Although a dynamic programming algorithm was described in Clote and Bayegan (2015) to compute the average $MS_2$ network degree, the methods of this paper do not easily generalize to $MS_2$ connectivity densities. Figure 11 shows a least-squares regression line for the log-log density plot for $MS_2$ connectivity (computed by brute-force) for a homopolymer of length 20, together with an optimal power-law fit computed by RNApowerlaw. Since there are only 106.633 secondary structures for the 20-mer with $\theta =3$, we ran powerlaw on $MS_2$ connectivity data, which determined $\alpha = 6.84$, $k_{{xmin}}=36$, and a log odds ratio $R= -2.06$ with *p* value of 0.248. Since RNApowerlaw determined $\alpha = 6.84$, $k_{{xmin}}=36$, and a Kolmogorov–Smirnov *p* value of 0.219, we can *not* reject the null hypothesis that a power-law distribution fits the tail of $MS_2$ connectivity data for a 20-mer.

## Conclusion

Since the pioneering work of Zipf on the scale-free nature of natural languages (Zipf 1949), various groups have found scale-free networks in diverse domains ranging from communication patterns of dolphins (McCowan et al. 2002), metabolic networks (Jeong et al. 2000), protein–protein interaction networks (Ito et al. 2000; Schwikowski et al. 2000), protein folding networks (Bowman and Pande 2010), genetic interaction networks (Tong et al. 2004; Van Noort et al. 2004) to multifractal time series (Budroni et al. 2017). These discoveries have galvanized efforts to understand biological networks from a mathematical and topological standpoint. Using mathematical analysis, Barabasi and Albert (1999) established that scale-free networks naturally emerge when networks are dynamic, whereby newly accrued nodes are preferentially connected to nodes already having high degree. On such grounds, one might argue that protein folding networks and protein–protein interaction (PPI) networks should exhibit scale-free properties, since nature is likely to reuse and amplify fast-folding domains—cf. Gilbert’s exon shuffling hypothesis (Gilbert 1978). Indeed, Cancherini et al. (2010) have established that in 4 metazoan species analyzed (*H. sapiens*, *M. musculus*, *D. melanogaster*, *C. elegans*) those genes, which are enriched in exon shuffling events, displayed a higher connectivity degree on average in protein–protein interaction (PPI) networks; i.e. such genes had a larger number of interacting partners. On similar grounds that nature should reuse and amplify successful metabolic networks, one might argue that metabolic networks should exhibit scale-free properties. However, rigorous statistical analysis has shown that metabolic networks fail a goodness-of-fit test for scale-free distribution, while PPI satisfy a goodness-of-fit test for scale-free distributions over a certain range of connectivity (Khanin and Wit 2006; Clauset et al. 2009).

**Fig. 12:**
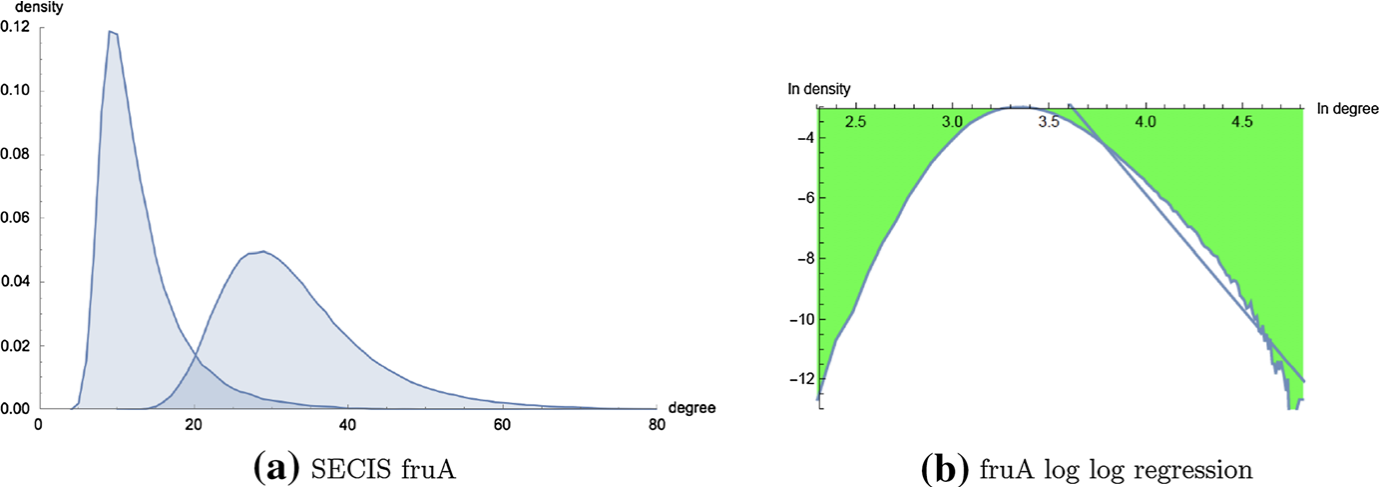
**a**$MS_1$- and $MS_2$-degree distribution for the 32 nt selenocystein insertion (SECIS) element fruA with sequence CCUCGAGGGG AACCCGAAAG GGACCCGAGA GG, obtained by brute-force computation from an enumeration of all secondary structures (exact number 971299), ranging in degree from 4 to 123. Average $MS_1$-degree 13.10; average $MS_2$-degree 33.25. Using notation from Table 9, the MLE power-law fit for $MS_1$-degree data has values of $k_{min} = 35$, $\alpha (35,123)=6.329$, $KS(35,123)=0.0221$, $\langle KS \rangle = 0.0075$, *p* value of 0.0000. In contrast, the MLE power-law fit for $MS_2$-degree data has values of $k_{min} = 93$, $\alpha (93,123)=14.441$, $KS(93,123)=0.0219$, $\langle KS \rangle = 0.0081$, *p* value of 0.729. Summarizing, Kolmogorov–Smirnov statistics indicate that the $MS_1$ data is *not* scale-free, while it cannot be refuted that the $MS_2$ data is scale-free. However, the range of degrees for which the $MS_2$ data might be scale-free is from 93 to 123, which accounts for only $5.77 \cdot 10^{-4}$ of the density. As shown in (**b**), even log-log regression suggests that the $MS_2$ data is *not* scale-free. **b** Log–log plot of $MS_2$-density of fruA with regression equation $\ln \text{ density } = 24.37 +7.56 \cdot \ln \text{ degree }$, determined from the relative frequency of structures having $MS_2$-degree in the range of 29 to 4123, corresponding to the portion of the $MS_2$ density starting after the peak of 0.04987 in previous panel at degree $k_{{peak}}=29$ (color figure online)

There appears to be a current polemic whether certain naturally occurring networks are scale-free. Broido and Clauset (2019) provide statistical arguments that less than 45 of the 927 real-world network data sets (i.e. $4\%$) found in the *Index of Complex Networks* exhibit the “strongest level of direct evidence for scale-free structure”. In a response to a preprint of Broido and Clauset (2019) dated March 6, 2018 and posted on the Barabási Lab web site https://www.barabasilab.com/post/love-is-all-you-need, A.L. Barabási argued against the conclusions of Broido and Clauset (2019). Here, it should be noted that this is not the first time a polemic has arisen about the power-law distribution—indeed, there was a heated exchange between Mandelbrot and Simon almost 60 years ago in the journal *Information and Control*. For details, references, and a history of the power-law distribution, see Mitzenmacher (2004).

**Fig. 13:**
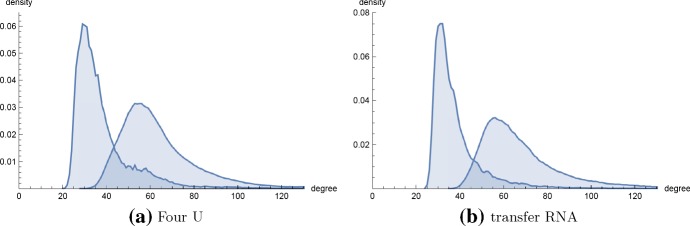
**a**$MS_1$- and $MS_2$-degree distribution for the 65 nt fourU RNA from *Klebsiella pneumoniae* subsp. pneumoniae with sequence GGACAAGCAA UGCUUGCCUU UAUGUUGAGC UUUUGAAUGA AUAUUCAGGA GGUUAAUUAU GGCAC and EMBL accession code CP000647.1/1773227-1773291. FourU RNA is a class of *thermometers* found in bacteria such as *E. coli*, *Salmonella*, *V. cholerae*, etc. that regulate protein expression by undergoing a conformation change triggered by temperature—for instance, the conformational change of the *V. cholerae* fourU thermometer at $37^{\circ }$C permits the transcription of a virulence factor. All 1,079,102 secondary structures having free energy within 13 kcal/mol of the minimum free energy (MFE) of this RNA were generated using RNAsubopt from the Vienna RNA Package (Lorenz et al. 2011). The $MS_1$ and $MS_2$ degree of each secondary structure were determined in order to produce the degree relative frequency histogram. Although the collection of structures having free energy within $13^\circ$C of the MFE contains over one million structures (computation required 1–2 days), there are 1995457849526533 ($\approx 1.99546 \times 10^{15}$) many secondary structures altogether. The average $MS_1$ degree is 38.0, while the average $MS_2$ degree is 64.2. **FourU**$MS_1$**analysis:** Using RNApowerlaw, $x_{\mathrm{min}}=93$, $\alpha =6.02$, and *p* value is 0 (to 10 decimal places). Using powerlaw, $x_{\mathrm{min}} = 96$, $\alpha = 6.02$, and the log ratio of power-law fit to log-normal fit is $R= -23.6283$ with corresponding *p* value of $1.77 \times 10^{-4}$—in other words, a log-normal distribution provides a significantly better fit than a power-law distribution for the $MS_1$ degree data of this fourU RNA. **FourU**$MS_2$**analysis:** Using RNApowerlaw, $x_{\mathrm{min}}=85$, $\alpha =6.159$, and *p* value is 0 (to 10 decimal places). Using powerlaw, $x_{\mathrm{min}} = 85$, $\alpha = 6.159$, and the log ratio of power-law fit to log-normal fit is $R= -122.1518$ with corresponding *p* value of $5.9389 \times 10^{-20}$—in other words, a log-normal distribution provides a significantly better fit than a power-law distribution for the $MS_2$ degree data of this fourU RNA. **b**$MS_1$- and $MS_2$-degree distribution for the 76 nt alanine transfer RNA from *Mycoplasma mycoides* with accession code tdbR00000006 from tRNAdb (Juhling et al. 2009) (accession code RA1180 from the Sprinzl tRNA database) with sequence GGGCCCUUAG CUCAGCUGGG AGAGCACCUG CCUUGCACGC AGGGGGUCGA CGGUUCGAUC CCGUUAGGGU CCACCA. All 408414 secondary structures having free energy within 13 kcal/mol of the minimum free energy of this RNA were generated using RNAsubopt from the Vienna RNA Package (Lorenz et al. 2011). The MS1 and MS2 degree of each secondary structure were determined in order to produce the degree relative frequency histogram. Although the collection of secondary structures having free energy within $13^\circ$C of the MFE contains about one-half million structures (computation required 1-2 days), there are 877346780605139050 ($\approx 8.77347 \times 10^{17}$) many secondary structures altogether. The average $MS_1$ degree is 38.1, while the average $MS_2$ degree is 68.3. **tRNA**$MS_1$**analysis:** Using RNApowerlaw, $x_{\mathrm{min}}=36$, $\alpha =5.1419$, and *p* value is 0 (to 10 decimal places). Using powerlaw, $x_{\mathrm{min}} = 36$, $\alpha = 5.1419$, and the log ratio of power-law fit to log-normal fit is $R = -95.3556$, with corresponding *p* value of $1.6193 \times 10^{-16}$—in other words, a log-normal distribution provides a significantly better fit than a power-law distribution for the $MS_1$ degree data of this fourU RNA. **tRNA**$MS_2$**analysis:** Using RNApowerlaw, $x_{\mathrm{min}}=114$, $\alpha =7.0845$ and *p* value is 0 (to 10 decimal places). Using powerlaw, $x_{\mathrm{min}} = 122$, $\alpha =7.1352$, and the log ratio of power-law fit to log-normal fit is $R=-41.1935$ with corresponding *p* value of $5.0374 \times 10^{-6}$—in other words, a log-normal distribution provides a better fit than power-law for the $MS_22$ degree data of this tRNA (color figure online)

**Table 4:** Table showing secondary structure preferential attachment probabilities

n	n+1	$S_{n}$	$S_{n+1}$	Succ	Fail	Succ/(Succ+Fail)	$\langle p(s',t'|s,t) \rangle$
5	6	2	4	5	1	83.33%	$0.8333 \pm 0.1667$
6	7	4	8	18	8	69.23%	$0.7222 \pm 0.4157$
7	8	8	16	90	37	70.87%	$0.7748 \pm 0.3260$
8	9	16	32	419	131	76.18%	$0.8105 \pm 0.2941$
9	10	32	65	1891	575	76.68%	$0.8122 \pm 0.2887$
10	11	65	133	7883	2498	75.94%	$0.8125 \pm 0.2891$
11	12	133	274	33,069	9763	77.21%	$0.8300 \pm 0.2730$
12	13	274	568	142,968	40,797	77.80%	$0.8322 \pm 0.2709$
13	14	568	1184	621,884	171,384	78.40%	$0.8366 \pm 0.2646$
14	15	1184	2481	2,723,993	723,887	79.00%	$0.8428 \pm 0.2587$
15	16	2481	5223	12,041,929	3,108,978	79.48%	$0.8478 \pm 0.2556$
16	17	5223	11,042	53,730,451	13,544,005	79.87%	$0.8518 \pm 0.2523$
17	18	11,042	23,434	241,738,083	59,258,399	80.31%	$0.8561 \pm 0.2485$
18	19	23,434	49,908	1,096,087,115	261,730,198	80.72%	$0.8598 \pm 0.2455$

The first two columns contain homopolymer length *n* and $n+1$, followed by the number of secondary structures in ${S}_n$ and ${S}_{n+1}$, then the total number of 4-tuples $(s,t,s',t')$ that succeed in demonstrating [resp. fail to demonstrate] preferential attachment, denoted by Succ [resp. Fail]. The next column contains the proportion Succ/(Succ+Fail) of 4-tuples that demonstrate preferential attachment, defined by Eq. (33), while the last column contains the expected preferential attachment $\langle p(s',t'|s)\rangle$, defined by Eq. (35). This expectation is obtained by computing the arithmetical average of the conditional probabilities $p(s',t'|s,t)$, defined by $p(s',t'|s,t) = P\left( dg(s') \ge dg(t') | dg(s) \ge dg(t), s \prec s', t \prec t' \right)$

Given the current interest in whether certain naturally occurring networks are scale-free, we have introduced a novel algorithm to compute the connectivity density function for a given RNA homopolymer. Our algorithm requires $O(K^2n^4)$ run time and $O(Kn^3)$ storage, where *K* is a user-specified degree bound $K \le \frac{(n-\theta )(n-\theta -1)}{2}$. Short of exhaustively listing secondary structures by brute-force, no such algorithm existed prior to our work. Since existent software appears unable to perform power-law fitting for exponentially large RNA connectivity data, we have also implemented code to compute and statistically evaluate the maximum likelihood power-law fit for an input histogram, using a very fast method to the density function of a sampled power-law distribution with given scaling parameter. Using the resulting code, called RNAdensity and RNApowerlaw, we have computed the connectivity density function for RNA secondary structure networks for homopolymers of length up to 150. Statistical analysis categorically shows that there is no statistically significant power-law fit for homopolymer RNA secondary structure network connectivity, despite the seemingly good visual fit shown in Fig. 9. Figure 12 shows that secondary structure network connectivity is not scale-free for the (real) 32 nt selenocysteine insertion sequence *fruA*. Figure 13 shows that the $MS_1$ and $MS_2$ degree distributions for other naturally occurring RNAs are not scale-free, in particular for the 65 nt RNA thermometer from *Klebsiella pneumoniae* subsp. pneumoniae with EMBL accession code CP000647.1/1773227-1773291 and the 76 nt alanine transfer RNA from *Mycoplasma mycoides* with accession code tdbR00000006 from tRNAdb Juhling et al. (2009) (accession code RA1180 from the Sprinzl tRNA database). While the density plot in Fig. 12 was produced by exhaustively enumerating all 971,299 secondary structures of the 32 nt *fruA*, Figure 13 was produced by enumerating all secondary structures having free energy within 13 kcal/mol of the minimum free energy, as computed by RNAsubopt from the Vienna RNA Package (Lorenz et al. 2011); this procedure generated 1,079,102 secondary structures (out of a total of $\approx 1.99546 \times 10^{15}$ structures) for the 65 nt fourU RNA, and 408,414 secondary structures (out of a total of $\approx 8.77347 \times 10^{17}$ structures) for the 76 nt tRNA.

Since (Day et al. 2003; Kihara and Skolnick 2003) have presented data that suggests that existent protein structures can be explained using only a small number of protein folds, we presented data in Table 4 that suggests that RNA secondary structures may satisfy a type of preferential attachment—a rigorous combinatorial argument establishes this fact for a modified notion of preferential attachment [data not shown, but available in the Appendix of Clote (2018)]. Finally, Python implementations of the algorithms from this paper are publicly available at http://bioinformatics.bc.edu/clotelab/RNAnetworks.

As an afternote, our personal opinion is that it doesn’t much matter whether a naturally occurring network arising from physical phenomena is precisely scale-free or not. If network connectivity appears to follow a power-law distribution, even approximately, then by results of Barabasi and Albert (1999), this suggests that preferential attachment could play a role in how the network may have been constructed by nature. Preferential attachment might well have been a factor in how protein and RNA structures have been formed by evolutionary forces—even in the emergence of stable folds in prebiotic times (Abkevich et al. 1997). It is noteworthy that only a small number of protein folds suffice to explain the diversity of all protein folds found in the Protein Data Bank (PDB) (Kihara and Skolnick 2003): “The number of proteins required to cover a target protein is very small, e.g. the top ten hit proteins can give 90% coverage below a RMSD of 3.5 Å for proteins up to 320 residues long.” As well, the 30 most populated metafolds represent “about half of a nonredundant subset of the PDB” (Day et al. 2003). However, other evolutionary factors seem to be present in the evolution of protein folds, such as kinetic accessibility (Cossio et al. 2010), as well as the ability to switch between alternate conformations (Porter and Looger 2018).

## Preferential attachment of RNA secondary structures

In this section, we provide preliminary data that suggest that *preferential attachment* holds in the homopolymer RNA secondary structure model. A rigorous argument can be found in the preprint (Clote 2018) for all homopolymer RNA networks, albeit with respect to a slight relaxation of our definitions. Before proceeding we recall basic definitions and notation. The notion of homopolymer secondary structure was defined at the beginning of Sect. 2.1; throughout this section, we denote the set of all secondary structures for a length *n* homopolymer by ${\mathcal {S}}_n$. If $s \in {\mathcal {S}}_{n}$ and $s' \in {\mathcal {S}}_{n+1}$, then we say that $s'$*extends**s*, and write $s \prec s'$, if $s'$ is obtained by either (1) appending unpaired nucleotide $n+1$ to the right of *s*, so that the dot-bracket notation of $s'$ is $s \bullet$, or (2) adding a base pair $(k,n+1)$ to *s*, where $k \in [1,n-\theta ]$ is *external* to every base pair of *s*, i.e. it is not the case that $i \le k \le j$ for any base pair (*i*, *j*) of *s*. Since the seminal papers of Stein and Waterman (1978), Nussinov and Jacobson (1980), this notion of extension has been used as the basis of recursive and/or dynamic programming algorithms to count/enumerate all secondary structures and to compute minimal free energy structures.

A reasonable approach to establish *preferential attachment* in the context of RNA secondary structures is to show that if the degree of *s* is greater than or equal to the degree of *t* in the network ${\mathcal {S}}_n$, then for most extensions $s'$ of *s*, and $t'$ of *t*, the degree of $s'$ is greater than or equal to the degree of $t'$ in the network ${\mathcal {S}}_{n+1}$. We show that this is indeed the case for homopolymers of modest length, using by brute-force, exhaustive computations in this section, and we rigorously establish this result for a relaxation ${\mathcal {S}}^*_n$ of the secondary structure model in Appendix A.

For fixed homopolymer length *n*, define the set ${\mathcal {A}}_n$ of 4-tuples $(s,t,s',t')$ by

32 $$\begin{aligned} {\mathcal {A}}_n&= \{ (s,t,s',t') : s,t \in {\mathcal {S}}_n, s',t' \in {\mathcal {S}}_{n+1}, s \ne t, s \prec s', t \prec t', dg(s) \ge dg(t) \} \end{aligned}$$

A 4-tuple $(s,t,s',t') \in {\mathcal {A}}_n$*succeeds* in demonstrating preferential attachment if $dg(s') \ge dg(t')$; otherwise the 4-tuple *fails* to demonstrate preferential attachment. Let Succ$_n$ [resp. Fail$_n$] denote the set of 4-tuples that succeed [resp. fail] to demonstrate preferential attachment, so that ${\mathcal {A}}_n = \text{ Succ }_n \cup \text{ Fail }_n$ (when *n* is clear, we drop the subscripts, and we ambiguously also use Succ and Fail to denote the sizes of these sets). Our first quantification of preferential attachment is given by the proportion Succ/(Succ+Fail):

33 $$\begin{aligned} P({Succ}_n)&= \frac{|\{ (s,t,s',t') \in {\mathcal {A}}_n: dg(s')\ge dg(t')\}|}{|{\mathcal {A}}_n|} \end{aligned}$$

Since secondary structures have possibly quite different degrees and numbers of extensions, a more accurate measure (in our opinion) of preferential attachment is given by $\langle p(s',t'|s,t) \rangle$, defined as follows. For distinct, fixed structures $s,t \in {\mathcal {S}}_n$, define

34 $$\begin{aligned} p(s',t' | s,t)&= P\left( dg(s') \ge dg(t') | dg(s) \ge dg(t), s \prec s', t \prec t' | dg(s) \ge dg(t) \right) \nonumber \\&= \left\{ \begin{array}{ll} 0 &{}\text{ if } \, dg(s)<dg(t) \\ \frac{ |\{ (s',t'): s',t' \in {\mathcal {S}}_{n+1}, s'\ne t', s \prec s', t \prec t', dg(s') \ge dg(t') \} |}{ |\{ (s',t'): s',t' \in {\mathcal {S}}_{n+1}, s'\ne t', s \prec s', t \prec t' \} |} &{}\text{ else } \end{array} \right. \end{aligned}$$

35 $$\begin{aligned} \langle p(s',t' | s,t) \rangle&= \frac{ \sum _{s,t \in {\mathcal {S}}_n, s\ne t} p(s',t'|s,t) }{ |\{ (s,t): s,t \in {\mathcal {S}}_n, s\ne t, dg(s) \ge dg(t) \} |} \end{aligned}$$

To clarify these definitions, we consider a small example. If $n=5$, then ${\mathcal {S}}_n$ consists of the two structures $\bullet \bullet \bullet \bullet \bullet$, and $\,{\texttt {(}}\,\bullet \bullet \bullet \,{\texttt {)}}\,$, while ${\mathcal {S}}_{n+1}$ consists of the four structures $\bullet \bullet \bullet \bullet \bullet \bullet$, $\,{\texttt {(}}\,\bullet \bullet \bullet \bullet \,{\texttt {)}}\,$, $\bullet \,{\texttt {(}}\,\bullet \bullet \bullet \,{\texttt {)}}\,$, $\,{\texttt {(}}\,\bullet \bullet \bullet \,{\texttt {)}}\,\bullet$. Fix *s* to be $\,{\texttt {(}}\,\bullet \bullet \bullet \,{\texttt {)}}\,$, and *t* to be $\bullet \bullet \bullet \bullet \bullet$. Since the only neighbor of *s* is *t*, and vice-versa, it follows that $dg(s)=1=dg(t)$. By definition, an extension $s'$ of *s* is obtained either by adding an unpaired nucleotide to *s* at position $n+1$, or by adding a base pair $(k,n+1)$ to *s*, where *k* is external to all base pairs of *s*. In the current case, the only possible extension of *s* is produced by the former rule, thus obtaining $s'=\,{\texttt {(}}\,\bullet \bullet \bullet \,{\texttt {)}}\,\bullet$. Note that we do *not* consider the structure $\bullet \,{\texttt {(}}\,\bullet \bullet \bullet \,{\texttt {)}}\,$ to be an extension of *s*. In contrast, the structure $t= \bullet \bullet \bullet \bullet \bullet$ has three extensions: $t'_1= \bullet \bullet \bullet \bullet \bullet \bullet$, $t'_2= \,{\texttt {(}}\,\bullet \bullet \bullet \bullet \,{\texttt {)}}\,$, $t'_3= \bullet \,{\texttt {(}}\,\bullet \bullet \bullet \,{\texttt {)}}\,$, where by definition, $t'_4= \,{\texttt {(}}\,\bullet \bullet \bullet \,{\texttt {)}}\,\bullet$ is not considered to be an extension of *t*. Clearly $dg(s') = dg(t'_2)$, $dg(s') = dg(t'_3)$, but $dg(s')=1 \not \ge dg(t'_1)=3$, so

$$\begin{aligned} \frac{2}{3}&= \frac{ |\{ (s',t'): dg(s') \ge dg(t') \wedge s \prec s', t \prec t', s,t \in {\mathcal {S}}_{n+1} \} |}{ |\{ (s',t'): s \prec s', t \prec t', s,t \in {\mathcal {S}}_{n+1} \} |} \end{aligned}$$

so $p(s',t'|s,t)= 0.6667$. If we now take $s = \bullet \bullet \bullet \bullet \bullet$, and $t = \,{\texttt {(}}\,\bullet \bullet \bullet \,{\texttt {)}}\,$, we find that

$$\begin{aligned} \frac{3}{3}&= \frac{ |\{ (s',t'): dg(s') \ge dg(t'), s \prec s', t \prec t', s,t \in {\mathcal {S}}_{n+1} \} |}{ |\{ (s',t'): s \prec s', t \prec t', s,t \in {\mathcal {S}}_{n+1} \} |} \end{aligned}$$

so $p(s',t'|s,t) = 1$. The (arithmetical) average of 1 and 2/3 is $\frac{2+3}{3} = 5/6 = 0.8333$, which is the value $\langle p(s',t'|s,t) \rangle$ found in the first row and last column of Table 4. In contrast to this value, averaged over all pairs $s,t \in {\mathcal {S}}_n$ for which $dg(s) \ge dg(t)$, the total number of *successes* [resp. *failures*] is 5 [resp. 1], where a success [resp. failure] is defined as a 4-tuple $(s,t,s',t')$ for which $s,t \in {\mathcal {S}}_n$, $s',t' \in {\mathcal {S}}_{n+1}$, $s \prec s'$, $t \prec t'$, $dg(s) \ge dg(t)$ and $dg(s') \ge dg(t')$ [resp. $dg(s')<dg(t')$]. Thus we find the value $5/6 = 0.8333$ in the first row and 7th column; however, it is not generally true that Succ$_n$/ (Succ$_n$+ Fail$_n$) agrees with $\langle p(s',t'|s,t) \rangle$, since *s*, *t* may have different degrees in ${\mathcal {S}}_n$, and each may have a different number of extensions $s\prec s'$, $t \prec t'$, and each $s',t'$ may each have different degrees in ${\mathcal {S}}_{n+1}$.

For homopolymers of length 5 to 18, Table 4 shows the proportion of successes, $P(\text{ Succ })$, defined in Eq. (33), as well as the average preferential attachment probabilities $\langle p(s',t'|s,t) \rangle$, defined in Eq. (35). Values in this table, produced by brute-force, exhaustive computation, were obtained for each homopolymer length $n \in [5,19]$, by first generating the collections ${\mathcal {S}}_n$, then computing the degrees *dg*(*s*) for $s \in {\mathcal {S}}_n$ by brute force, then considering all ${n \atopwithdelims ()2}$ unordered pairs *s*, *t* of distinct structures in ${\mathcal {S}}_n$. So far, the number of instances to consider is large—for instance, when $n=18$, there are ${n \atopwithdelims ()2} = 274,564,461$ unordered pairs of distinct structures from ${\mathcal {S}}_n$. For each pair of distinct structures *s*, *t* from ${\mathcal {S}}_{n}$ that satisfy $dg(s) \ge dg(t)$, a list $L_s$ [resp. $L_t$] of extensions $s \prec s'$ [resp. $t \prec t'$] were computed, where the size of each list is one plus the number of positions in $[1,n-\theta ]$ that are external to every base pair of *s* [resp. *t*]. Subsequently, the proportion of extension pairs $s',t'$ that satisfy $dg(s') \ge dg(t')$ is determined, thus yielding $p(s',t'|s,t)$. Finally, the mean and standard deviation of the latter yields $\langle p(s',t'|s,t) \rangle$, shown in the last column of the table. For $n=18$, more than one trillion ($1.36 \cdot 10^9$) 4-tuples $(s,t,s',t')$ where considered for which $dg(s) \ge dg(t)$—this value is used in the denominator of Eq. (35)!

From the values in Table 4, it appears that the RNA homopolymer secondary structure model does demonstrate preferential attachment. This, in our opinion, may provide theoretical justification for the close approximation of the tail of degree distributions by a power-law distribution, even though a rigorous statistical test by bootstrapping Kolmogorov–Smirnov values solidly rejects this hypothesis.

## Footnotes

1 A network is said to be *robust*, or *resilient*, if its connectivity is (relatively) unaltered in the event that random nodes have been removed; i.e. alternate pathways exist to connect nodes, even if a (random) node has been removed. Since functionality remains in the case of random node failure, network robustness is of obvious importance in massively parallel computers, in the World Wide Web, in metabolic pathways, signaling pathways, etc. This topic is discussed in detail in Chapter 16, “Percolation and Network Resilience”, in Newman (2010).
